# One-Pot Deconstruction and Conversion of Lignocellulose Into Reducing Sugars by Pyridinium-Based Ionic Liquid–Metal Salt System

**DOI:** 10.3389/fchem.2020.00236

**Published:** 2020-04-15

**Authors:** Sadia Naz, Maliha Uroos, Azmat Mehmood Asim, Nawshad Muhammad, Faiz Ullah Shah

**Affiliations:** ^1^Institute of Chemistry, University of the Punjab, Lahore, Pakistan; ^2^Interdisciplinary Research Centre in Biomedical Materials (IRCBM), COMSATS University Islamabad, Lahore, Pakistan; ^3^Chemistry of Interfaces, Luleå University of Technology, Luleå, Sweden

**Keywords:** lignocellulosic biomass, ionic liquids, lewis acidic catalyst, cellulose, lignin, total reducing sugars, one-pot, deconstruction

## Abstract

Constantly decreasing fossil resources and exceeding energy demands are the most alarming concerns nowadays. The only way out is to develop efficient, safe, and economical biomass processing protocols that can lead toward biofuels and fine chemicals. This research is one of such consequences involving the deconstruction and conversion of wheat straw carbohydrate constituents into reducing sugars via one-pot reaction promoted by Lewis acidic pyridinium-based ionic liquids (PyILs) mixed with different metal salts (MCl). Various parameters such as the type of metal salt, loading amount of metal salt, time, temperature, particle size of biomass, and water content which affect the deconstruction of wheat straw have been evaluated and optimized. Among the studied ionic liquid (IL) and metal salt systems, the best results were obtained with [BMPy]^+^${\text{CoCl}}_{3}^{-}$. The dinitrosalicylic acid (DNS) assay was used to determine the percentage of total reducing sugars (TRS) generated during treatment of wheat straw. The deconstructed wheat straw was characterized with various analytical tools, that is, Fourier transform infrared (FTIR) spectroscopy, scanning electron microscopy (SEM), and X-ray powder diffraction (XRD) analyses. The IL–metal salt system was recycled for subsequent treatment of wheat straw. Statistical parameters were calculated from analysis of variance (ANOVA) at the 0.05 level of confidence.

## Introduction

Lignocellulosic biomass is the most economic and abundant primary energy resource with a worldwide production of about 1 × 10^10^ million tons per annum (Alvira et al., 2010). It is the only renewable and promising carbon source in the scenario of the world's critical “bioenergy” demand, alarming climate concerns, and various other downsides of constantly depleting fossil fuel non-renewable resources (Brandt et al., 2013; Zhang et al., 2016). It is predicted that the chemical industry will rely on it for almost 30% of its total raw materials after a decade (Alvira et al., 2010). To achieve this claim, the so-called “integrated biorefineries” are trying to develop efficient routes for biomass transformation into energy, valuable platform chemicals, and biofuels providing an alternative to petrochemicals and petrofuels (Brandt et al., 2013).

Lignocellulosic biomass is a composite material containing polysaccharides such as cellulose and hemicellulose and polymeric aromatics such as amorphous phenylpropanoid lignin. Moreover, less percentage of proteins, pectins, inorganic compounds, and extractives such as waxes and lipids is also present. To defeat this structural complexity, pretreating the lignocellulosic biomass is an imperious task for setting up biomass-derived carbohydrate chemistry. A number of approaches such as physical (da Silva et al., 2010), chemical (Hsu et al., 2010), physicochemical (Varga et al., 2004), and biological (Wan and Li, 2010) pretreatments or their combination (Zhu et al., 2006; Alinia et al., 2010; Ma et al., 2010) have been employed significantly. Traditional methods employed so far involve harsh conditions such as high temperature and pressure, making the processes economically less feasible. The ionic liquid (IL) pretreatment approach overtakes all these conventional processes because ILs are greener, economical, and non-degradative for lignocellulose (Moyer et al., 2018). ILs tend to produce high monomeric sugars starting from biomass and prevent them from degradation. They also limit the inhibitory products and carbon dioxide formation, solvent loss, and consumption of energy (Rocha et al., 2017). They also strongly favor deconstruction to ramify lignocellulosic biomass into lignin and carbohydrates as it is a crucial step in biomass-derived carbohydrate chemistry in order to get products from all the biomass components. The cellulosic framework of lignocellulose can be hydrolyzed into reducing sugars via cleavage of biopolymeric β-1,4-glycosidic bonds, thus unlocking the potential of cellulose for further processing (Yoo et al., 2017). Contrarily, the other major lignocellulosic aromatic component allows the formation of wide-ranging bulk and fine aromatic compounds due to its matchless chemical and structural properties. Although it has been considered less, it is thought to be a major aromatic resource of the bio-based economy for production of biofuels and fine chemicals (Zakzeski et al., 2010). In short, the pretreatment using ILs is economical and beneficial for reduction of energy demands.

ILs-based pretreatment of lignocellulosic biomass has been coupled with various catalytic systems such as solid acid (Chen et al., 2018), Brønsted acid (Suzuki et al., 2018), and soluble Lewis acid catalysis (Eminov et al., 2016) to afford better results. Among these, the Lewis acid catalytic system is considered the most efficient for biomass conversion to fine chemicals and was first ascribed by Zhao et al. (2007) a decade ago. Later on, a number of reports have been made to explore the catalytic ability of several metal salts as soluble Lewis acids for saccharification of cellulose and subsequent 5-hydroxymethylfurfural (5-HMF) production that is among the most important biomass-derived chemicals (Qu et al., 2016) and has been recognized as a promising renewable platform chemical by the U.S. Department of Energy (Bozell and Petersen, 2010). A wide range of Lewis acids have been tested to date, with the best ever results with halides of chromium (Binder and Raines, 2009; Song et al., 2013), aluminum (Xiao et al., 2014), and iron (Tao et al., 2010). The role of these metal salts is to enhance the saccharification of cellulose to produce monosaccharides such as glucose and its isomerization to fructose followed by dehydration to yield 5-HMF. In contrast, low yields are associated with mineral acids and other solid acids probably due to the inefficiency of these catalysts to isomerize glucose to fructose (Wang et al., 2011). Also, they just facilitate the cleavage of the glycosidic linkage of the carbohydrate polymers (Zhang and Zhao, 2010).

In this context, this work aimed to develop a stairway to effectively utilize all the lignocellulosic contents in integrated biorefineries for effective production of biofuels and fine chemicals by using ILs as efficient pretreatment media. Besides all the advantages and green profile of ILs, the only downside in commercializing ILs in integrated biorefineries is their high cost. This is due to the expensiveness of their starting reagents. Thus, the only solution is to use low-cost materials for synthesis and to use the resultant ILs in biomass processing (Asim et al., 2019). Another way to enhance process efficiency for large-scale experimentation is to develop a one-pot process using cheap ILs such as protic ones for less operating and capital costs (Xu et al., 2016). In this study, we have used pyridinium-based ILs (PyILs) that are reported to be four times cheaper than the extensively studied imidazolium ones. The synthesis method and process cost are almost the same as those for imidazolium ILs. They can be a promising substitute of imidazolium ILs due to their favorable ecotoxicological profile; they are less toxic and mineralize or decompose faster than imidazolium ones (Sashina et al., 2016). To the best of our knowledge, this is the first report on the use of a PyIL/MCl system for biomass deconstruction/delignification and its simultaneous transformation into key chemical intermediates. Contrarily, there exist various reports for conversion of lignocellulosic biomass by imidazolium ILs, but most of them pay no attention to valuable lignin component and focus only on cellulose saccharification (Cheng et al., 2016; Yu et al., 2018). The reports exhibiting deconstruction along with saccharification follow a two-step protocol; at first, IL is used to separate lignin from cellulose, and then acids, alkalis, or enzymes are applied on the extracted delignified cellulose to yield sugars (Kassaye et al., 2017; Rocha et al., 2017; da Costa Lopes et al., 2018; Semerci and Guler, 2018). Some recent reports revealed the simultaneous removal of lignin and sugars from biomass using Brønsted acidic ILs but at high temperatures: 120–170°C (Gschwend et al., 2012; Chambon et al., 2018).

From our previous research finding, the PyIL 1-butyl-3-methylpyridinium chloride ([BMPy]Cl) was chosen due to its economical synthesis and significant results with microcrystalline cellulose (MCC), 28 wt% dissolution, and 78% total reducing sugars (TRS) (Saher et al., 2018). This IL was made acidic by mixing with Lewis acids (metal salts) so that it can effectively interact with wheat straw constituents for maximum generation of TRS that has been measured by dinitrosalicylic acid (DNS) assay. The process of biomass treatment has been optimized with respect to metal salt types, loading amount of metal salt, temperature, time, particle size of biomass, and water content. Various analytical characterizations, that is, Fourier transform infrared (FTIR) spectroscopy, scanning electron microscopy (SEM), and X-ray powder diffraction (XRD), were used to investigate the effect of the PyIL/MCl system on the wheat straw structure and further confirmation of fractionation process. The stability and recycling of PyIL were confirmed through nuclear magnetic resonance (NMR) spectroscopy.

## Experimental

### Materials and Methods

3-Methylpyridine (Merck Schuchardt), 1-chlorobutane (Fisher Scientific), 3,5-DNS (Sigma Aldrich), and metal chlorides were of analytical grade and used without any further purification. Solvents such as ethyl acetate and *n*-hexane were purchased and distilled prior to use. Wheat straw as lignocellulosic feedstock was harvested from a local farm in Lahore, Punjab, Pakistan, ground and meshed to collect particle sizes of 100, 250, and 500 μm for investigation. The samples were dried at 70°C for 48 h to remove moisture before processing in ILs. The wheat straw contained 30% cellulose, 44% hemicellulose, 15% lignin, 3.8% ash, 8.5% moisture, and 2% extractives as calculated according to Plazonic et al. (2016).

Progress of IL synthetic reaction was monitored via TLC (DC-Alufolien silica gel 60 F_254_ Merck) and visualized under a UV lamp (UVGL-25 minor light multiband UV-254/366). TRS content produced was quantified by DNS assay using a UV–spectrophotometer (UVD-3500, Labomed Inc., USA). Spectroscopic analyses by FTIR and ^1^HNMR were recorded via Agilent Cary 630 FTIR with a scanning range of 4,000–400 cm^−1^ and Advance AV at 400 MHz, respectively. SEM was done via SEM Vega Tescon with variable pressure. Samples were made conductive by gold sputtering, and images were taken at different magnifications. The change in crystallinity due to delignification was recorded using PAN analytical X′pert PRO diffractometer using CuK_α_ radiation (λ = 1.54 Å) at 40 kV and 30 mA with a scanning rate of 0.01°/s and scanning speed of 1°/min in a 2θ range of 10–80°.

### Synthesis of 1-Butyl-3-Methylpyridinium Chloride

The IL 1-butyl-3-methylpyridinium chloride [BMPy]Cl was prepared according to literature (Heinze et al., 2005) with a slight modification. In a 100-ml round-bottom flask, 1 eq. of 3-methylpyridine and 1.5 eq. of 1-chlorobutane were added and refluxed at 110°C with continuous stirring at 120 rpm. After some time, white solids began to form, indicating the successful initiation of reaction. Progress of reaction was monitored via TLC using 50% ethyl acetate: *n*-hexane solvent system. Reaction was continued to 35–40 h until the formation of a considerable amount of product. After that, the product was washed several times with ethyl acetate to ensure the complete removal of unreacted 3-methylpyridine. The final single spotted pure IL was stored in an oven-dried vial and sealed to prevent moisture.

White solid, yield (85%), MP (95–97°C), literature MP (95°C) (Heinze et al., 2005).

FTIR (cm^−1^): 3,058 (Ar–H stretching), 2,962 (C–H stretching of alkanes), 1,633 (C = N), 1,505 (C = C), 1,157 (C–N).

^1^HNMR (400 MHz, D_2_O) δ (ppm): 0.85 (3H, t, *J* = 7.6 Hz, CH_3_), 1.24–1.29 (2H, m, CH_2_), 1.88–1.93 (2H, m, CH_2_), 2.46 (3H, s, CH_3_), 4.83 (2H, t, *J* = 7.2 Hz, CH_2_), 7.85 (1H, t, *J* = 6.8 Hz, Ar–H), 8.27 (1H, d, *J* = 8 Hz, Ar–H), 8.56 (1H, d, *J* = 5.6, Ar–H), 8.62 (1H, s, Ar–H) (Figure S2).

### Lignocellulosic Biomass Processing

#### General Processing Protocol

[BMPy]Cl (0.9g) was taken in a glass vial immersed in an oil bath and stirred constantly at 100°C for 10 min to ensure the removal of all possible moisture content. To this molten salt, metal chloride (10 wt%) was added and stirred for 30 min followed by the addition of wheat straw (10 wt% of total mass M_0_). Reaction mixture was heated with constant stirring at a specified reaction temperature for a considerable time. After that, a 20 mg sample was withdrawn for sugar analysis.

#### Reducing Sugar Analysis

Percentage of TRS was measured by DNS assay as reported by Zhou et al. (2013). The sample withdrawn after the saccharification process was diluted 150 times, and 1 ml of DNS solution was added in it. The resultant solution was heated in boiling water for 15 min, and color intensity of the solution was measured at 540 nm using a UV–vis spectrophotometer (Ramli and Amin, 2014). TRS concentration was calculated by using the calibration curve of standard glucose solution (Figure S1). The percentage of TRS was calculated according to the following equation (Wang et al., 2014):

(1) $$\begin{array}{l} {\text{Y}}_{\text{TRS}}=\;\frac{{\text{C}}_{\text{TRS}}\times {\text{M}}_{2}\times \;150}{{\text{M}}_{\text{b}}\times 1.11\times \frac{{\text{M}}_{2}}{{\text{M}}_{0}}}\;\times 100\text{ \%} \end{array}$$

where C_TRS_ (g/L) is the concentration of TRS, M_2_ (g) is the mass of sample withdrawn from the reaction mixture, M_b_ (g) is the mass of wheat straw added to the reaction, M_0_(g) is the total mass of the reaction mixture, 150 is in multiples of diluted sample, and 1.11 is the ratio of molecular weight of sugar C_6_H_12_O_6_ to that of cellulosic content C_6_H_10_O_5_ of wheat straw.

#### Biomass Fractionation

After saccharification reaction, antisolvents (equal volumes of deionized water and acetone) (Sun et al., 2009) were added to the reaction mixture containing possibly the cellulose, lignin, reducing sugars, and other fine chemicals. Water separated the cellulose from IL, thus causing it to regenerate. Acetone helped in the separation of lignin. Regenerated cellulose-rich material (CRM) was collected via filtration and washed several times with water. Filtrates containing IL, water, acetone, and lignin were subjected to acetone evaporation followed by centrifugation for lignin separation.

#### Regeneration and Recycling of IL

After lignin removal, residual aqueous phase was subjected to heat in order to ensure complete removal of water to regenerate the IL–metal salt catalytic system. Percentage of the recovered IL was calculated on the basis of the initial amount of IL used for the reaction.

(2) $$\begin{array}{l} \text{Recovered IL }\left(\%\right)\text{=}\frac{\text{weight of dried recovered IL}}{\text{weight of iniatially used IL for reaction}}\;\times \text{100} \end{array}$$

This regenerated system was used in the next runs by adding fresh wheat straw under the same reaction conditions, and recyclability of the system was noted.

#### Experimental Designs

All the experiments were performed in triplicates, and the effects of independent variables, type of metal chloride and their different catalytic loadings, biomass loading into IL, time, temperature, biomass particle size, and water content were studied. From all these independent variables, effect of quantitative ones (catalyst loading X_1_, reaction time X_2_, temperature X_3_, and biomass particle size X_4_) on the response (TRS production, percentage conversion, and delignification) was analyzed statistically to determine their mean ± SD. Analysis of average, standard deviation, and standard error was performed using IBM SPSS Statistics 21. One-way analysis of variance (ANOVA) was also performed by IBM SPSS Statistics 21 and OriginPro 8.5 at 0.05 level to determine the significance of differences between the response and independent variables (see Table S1). Results show that population means are not significantly different for all the factors under all independent variables. Exception was observed for TRS factor with X_3_ variable. The coefficients of determination (*R*^2^ values: 0.71022, 1, 0.08705, and 0.07469) also predict the goodness of fit on the data.

## Results and Discussion

### Lignocellulosic Biomass Processing

**Figure 1:**
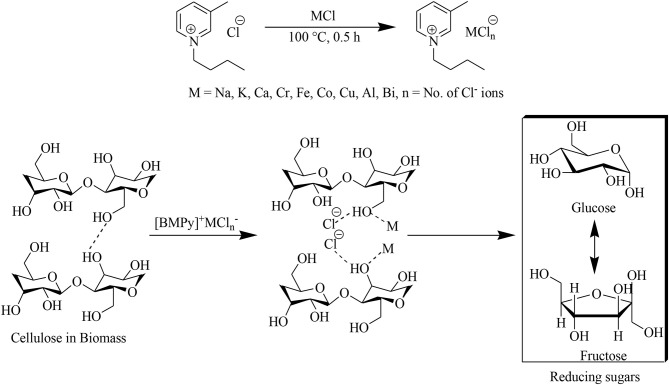
Metal chlorides associated formation of reducing sugars from lignocellulosic biomass.

The pre-dried ionic liquid [BMPy]Cl when heated and stirred with a catalytic amount of metal chlorides is predicted to form an IL/MCl complex (Zhao et al., 2007) in the form of [BMPy]^+^MCl_*n*_^−^. In this complex, wheat straw was processed for a suitable time and temperature to separate its cellulose and lignin contents as well as to saccharify the cellulose. Interaction of lignocellulosic wheat straw with this Lewis acidic IL–metal complex is facilitated by disruption of the hydrogen bonding network present in cellulose (Figure 1) as well as n–π and π-π conjugation, dispersive, and ion–dipole interactions for lignin dissolution (Pang et al., 2016).

### Effect of Process Variables

The influence of various reaction parameters like the type of metal chlorides and their different catalytic loadings, biomass particle size, water content, time, and temperature was monitored for optimization of one-pot delignification and simultaneous conversion of wheat straw into reducing sugars. Biomass loading into the IL was optimized by carrying out the reaction without any metal chloride with the result that 10 wt% is best with respect to maximum deconstruction and TRS production.

#### Optimization of Metal Chlorides

ILs were made Lewis acidic by using nine different salts containing chlorides of various metals ranging from alkali and alkaline earths to *d*-block transitions as well as some *p*-block non-metals. Of these Lewis acids, 10 wt% was added to IL to check their efficiency for delignification and carbohydrate saccharification (Table 1).

**Table 1:** Optimization of metal chlorides.

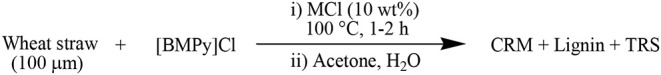					
**Sr. No**.	**MCl*_***x***_***	**TRS (%)**		**% Conversion^**a**^**	**% Delignification^**b**^**
		**1 h**	**2 h**		
1	None	45	43	12	<1
2	NaCl	20	15	5	<1
3	KCl	15	23	8	<1
4	CaCl_2_	34	21	7	<1
5	CrCl_3_	61	60	28	20
6	FeCl_2_	60	47	23	75
7	CoCl_2_	65	70	32	67
8	CuCl_2_	39	21	8	5
9	AlCl_3_	66	41	29	12
10	BiCl_3_	35	32	14	5

All experiments were carried out with 10 wt% biomass loading.

a Percentage conversion of carbohydrate content of wheat straw calculated by the difference between actual and regenerated contents (Abou-Yousef and Steele, 2013) (moles of reacted cellulosic content/moles of initial cellulosic content) × 100%.

b *With respect to the actual lignin content of wheat straw (Weerachanchai and Lee, 2013); extracted lignin content (wt%) = (mass of extracted lignin/mass of lignin in raw biomass) × 100*.

Experiments were carried out at 100°C, and samples for TRS analysis were collected at two different time intervals: after 1 and 2 h. Percentage of TRS was calculated by using DNS assay while measuring the absorbance at 540 nm. Actually, the absorbance is due to the brown-colored 3-amino-5-nitrosalicylic acid (ANS) that is formed by redox reaction of 3,5-DNS with sugars (Yuan et al., 2016). All Lewis acids show maximum TRS after 1 h of processing except for hexahydrated cobalt chloride that showed the highest conversion of cellulosic content of wheat straw after 2 h of processing. This maximum efficiency of cobalt chloride is probably due to its greater coordination tendency to form a stable complex with [BMPy]Cl, as the role of these metal chlorides is to make a Lewis acidic complex by coordinating with the anion of IL that in turn enhances the saccharification of cellulose to produce monosaccharides such as glucose and fructose by cleaving the biopolymeric β-1,4-glycosidic bonds. On the other hand, maximum delignification was associated with FeCl_2_ despite the considerable lignin extraction (67 %) associated with CoCl_2_·6H_2_O.

Addition of metal chlorides was of pronounced worth as 20 and 75% respective increments in conversion and delignification rates were obtained when compared with the reactions lacking these. All metal chlorides were not equally effective; for example, BiCl_3_ and chlorides of alkali and alkaline earths did not work. Exceptional behavior was that of CuCl_2_, which did not form a complex with IL. Paired metal catalytic systems CoCl_2_/CrCl_3_ and CoCl_2_/AlCl_3_ were also tested but no significant results were observed. As the CoCl_2_·6H_2_O-catalyzed pretreatment was the most effective, it was used for screening of further parameters.

#### Optimization of Catalytic Loading of MCl_x_

**Figure 2:**
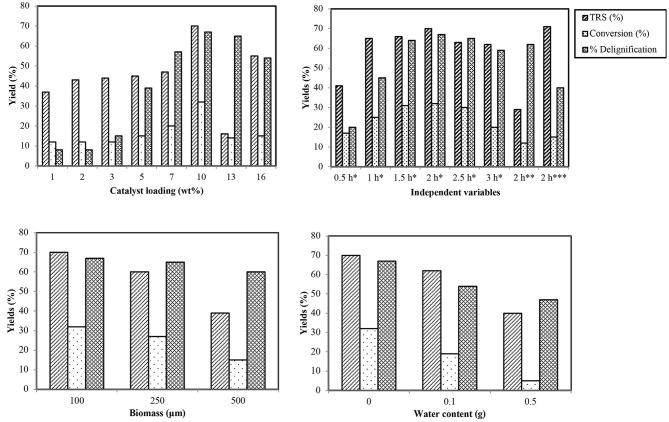
Effect of different variables on TRS, conversion, and delignification percentage. *100°C, **80°C, ***120°C.

Encouraged by the highest catalytic efficiency of CoCl_2_·6H_2_O, screening of its different wt% loadings in IL was done for best pretreatment results. Starting from a unit wt%, catalytic loading was gradually increased with the outcome that by increasing it, saccharification and delignification rates were enhanced up to a certain limit. The highest degradation profile was observed for 10 wt%; after that, increasing the loading caused a sudden decrease in percentage of TRS and lignin extraction (Figure 2). These results can be justified by considering the initial amount of IL used in the reaction mixture for which 10 wt% is the maximum loading for acceleration of reaction. Too much catalyst present in the reaction also leads to some side reactions.

#### Study of Time Effect

Time influence was monitored by carrying out the reactions at 100°C with optimum catalyst at regular intervals of 30 min each. Results (Figure 2) revealed that the rate of depolymerization of wheat straw is directly related to time, and the best results were obtained after 2.0 h. After that, a sharp TRS diminution takes place, which may be due to certain other side reactions and by-products.

#### Effect of Temperature

After the optimum time has been identified, temperature profile of the reaction was studied by carrying out trials at 80, 100, and 120°C. Rate of reaction was observed to increase linearly with increase in temperature up to 100°C; after that, it tends to decrease, causing the charring of the reaction mixture (Figure 2). The reason behind the trend of reaction acceleration and deceleration with respect to temperature is the reduction of viscosity of the reaction system at high temperature that increases the mass transfer rate, and thus, the cellulose chain in wheat straw biomass is degraded easily (Zhou et al., 2013). Decrease in percentage after optimal temperature is attributed to the instability of products and acceleration of sideways reactions like polymerization of TRS and HMF forming certain other by-products such as dimers of furan and humins (Sievers et al., 2009). High temperature also causes more energy consumption, making the process uneconomical. Hence, the suggested most suitable temperature for this reaction is 100°C with optimized time and catalyst.

#### Effect of Biomass Particle Size

A small particle size of biomass is required for processing so that viscous IL can be better penetrated and diffused into the interior of cell walls due to its composite composition (Pang et al., 2016). Size is mainly reduced by grinding the biomass, which causes a rise in consumption of cost and energy, causing the processing to be a little more uneconomical. To check the effect of biomass particle size, wheat straw with three different sizes (100, 250, and 500 μm) was processed in IL under optimized conditions of time, temperature, and metal salts. The results showed an inverse relation of high biomass particle size with dissolution and deconstruction of biomass as is apparent by less percentage conversion of wheat straw cellulose (Figure 2).

#### Effect of Water

Lignocellulosic biomass processing is highly sensitive to moisture content. As ILs exhibit high water affinity and biomass can also absorb moisture from air, avoiding the process in which water content enters the system is a critical step as it reduces the solvating power of the anion by binding strongly with its anion. That is the reason of incomplete biomass destruction causing reduced depolymerization of biopolymeric components in the presence of water (Zhou et al., 2015). The effect of water on conversion and deconstruction of biomass was studied by adding different amounts of water into the reaction under optimized reaction conditions (Figure 2). The percentage of sugar content and deconstruction was noted to be reduced by increasing the amount of water.

### Regeneration Process and Biomass Fractionation via Anti-Solvents

Fractionation of lignocellulosic biomass mainly relies on the careful selection of an antisolvent that is the most critical factor in the regeneration of lignin as well as cellulose-rich content of biomass. The most frequently reported antisolvents are water, acetone, and alcohols. Enzymatically saccharified biomass is usually regenerated via water, while lignocellulosic biomass pretreated with ILs are fractionated via water along with some organic solvents such as methanol, ethanol, acetone, and dimethyl sulfoxide (Ogura et al., 2014).

In this study, we used equimolar water and acetone as antisolvent to precipitate dissolute wheat straw. Cellulose-rich delignified content was regenerated by the action of water that critically disturbs the strong association between IL and cellulosic content of the lignocellulosic biomass. While lignin recovery is due to acetone that acts as a proficient medium to effectively dissolve all the lignin content of the pretreated biomass and later on regenerate it by evaporating itself. Filtration was performed to separate the regenerated cellulose-rich material (CRM), while the lignin was recovered by acetone evaporation from the filtrate followed by centrifugation. The retrieved lignin fraction was approximately 75 wt% of the original lignin content or 13 wt% in terms of original biomass content. As lignin is the main obstacle for utilization of carbohydrate content of biomass (Mosier et al., 2005), its isolation from biomass is an imperative task for developing integrated biorefineries. If successfully carried out, it improves the enzymatic hydrolysis of carbohydrates, and thus, cellulosic material of biomass can better be utilized.

### Material Balance

Material balance was calculated based upon the obtained masses of regenerated cellulose-rich material and lignin. Starting from 100 mg biomass, 50 mg of CRM and 13 mg of lignin were obtained, which account for a net total of 63 mg. The remaining 37 mg is attributed to the reducing sugars formed during the course of the reaction, and a little mass may correspond to loss of extractives/ash present in biomass.

### Characterization of Delignified Biomass and Lignin Samples to Confirm Fractionation

#### FTIR Analysis

**Figure 3:**
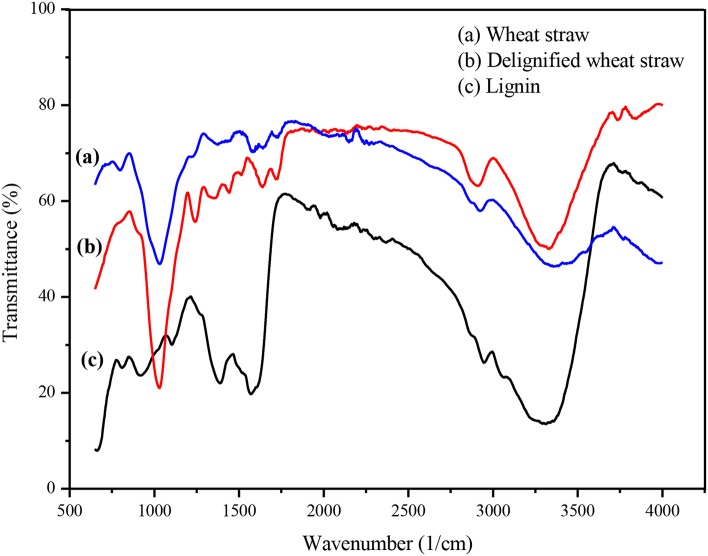
FTIR of wheat straw before and after processing.

Untreated and delignified wheat straw and extracted lignin were subjected to FTIR spectroscopic analysis to confirm the process of fractionation. In the FTIR spectra of wheat straw, a number of peaks were related to cellulose and lignin. After processing, the FTIR results showed refinement of cellulose and lignin peaks (Figure 3). The IR spectrum of the regenerated CRM exhibits peaks responsible for the hydroxyl group (3,329 cm^−1^); methyl, methylene, and methyne groups (2,891 and 1,635 cm^−1^); and diaryl C–O stretch and alkyl–aryl (R–O) stretch (1,240 and 1,029 cm^−1^) (Tao et al., 2011), while the FTIR spectrum of lignin contains characteristic peaks in the aromatic region confirming the fractionation process. Aromatic skeletal vibration breathing with C=O stretching is observed at 1,562 cm^−1^, whereas aromatic skeleton C–C stretching is obvious at 1,383 cm^−1^ (Ogura et al., 2014).

The effect of the different metal chlorides on fractionation was also studied by FTIR spectroscopy of the respective CRM (Figure S4) and lignin samples (Figure S5). The obtained results were in complete accordance with those described before (Section Optimization of Metal Chlorides); FTIR spectra showed sharp cellulosic and lignin peaks with chlorides of cobalt, aluminum, chromium, and iron, declaring alkali and alkaline earths and BiCl_3_ to be totally ineffective.

#### XRD Analysis

**Figure 4:**
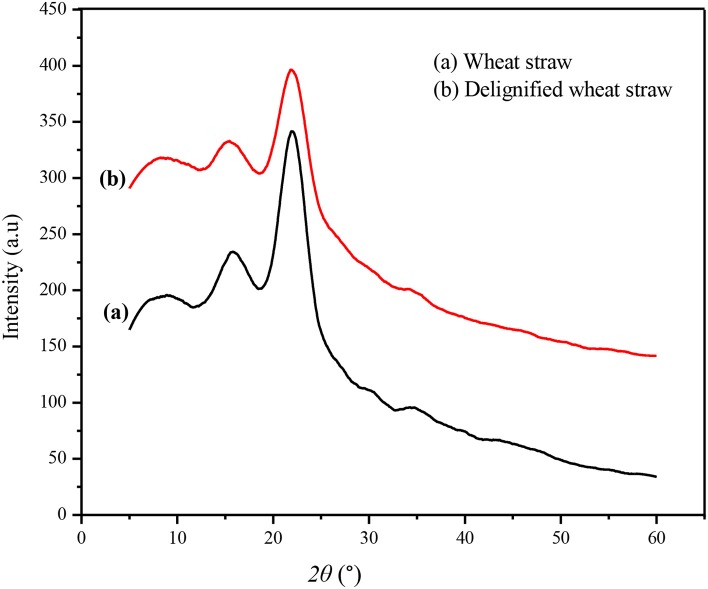
XRD analysis of untreated and delignified wheat straw.

X-ray diffraction spectra of the untreated and IL-pretreated wheat straw are shown in Figure 4. Untreated wheat straw showed the main peaks of crystalline cellulose at 22° and 15° while wheat straw pretreated with IL presented less X-ray diffraction intensity, confirming the efficiency of the IL/MCl system to destroy the cellulose crystalline structure. Thus, the regenerated CRM is less crystalline and is easily accessible to enzymatic saccharification and fermentation for bioethanol production or conversion into other value-added products.

#### SEM Analysis

**Figure 5:**
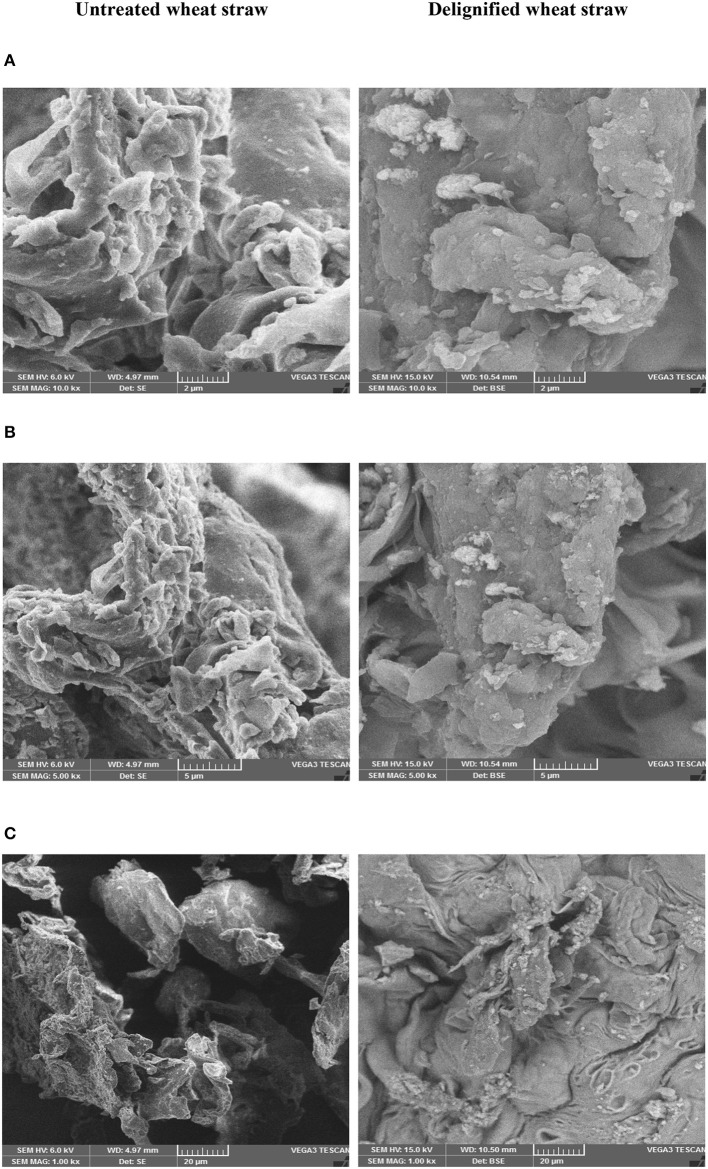
SEM images of untreated and delignified wheat straw **(A)** 2, **(B)** 5, and **(C)** 20 μm.

The effect of IL pretreatment on morphology and structure of wheat straw was noted in SEM images taken at different magnifications (Figures 5A–**C**). The untreated wheat straw appeared as rough flakes exhibiting highly rigid structure due to the presence of lignin and its compact binding with that of cellulose due to the cohesiveness imparted by hemicellulose. On the other hand, delignified wheat straw showed different morphology, having a highly porous flat sheet with a rough surface and thus making the enzymatic saccharification more favorable by allowing deeper penetration of enzymes.

#### Recycling Experiments

**Figure 6:**
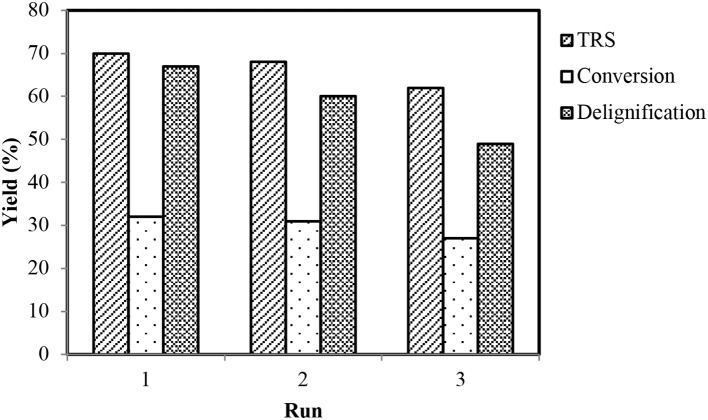
Efficiency of recycling experiments.

The IL–metal catalytic systems showed greater regeneration (about 86% recovery for [BMPy]^+^${\text{CoCl}}_{3}^{-}$) and recycling abilities. Regeneration was carried out by evaporation of water from the remaining filtrate after lignin extraction. This regenerated IL (viscous liquid with specific metal coloration) was confirmed via ^1^HNMR (Figure S3) and FTIR (Figure S6) analyses and was recycled by addition of fresh wheat straw in the next run. The best recycling ability was observed as is obvious by considerable conversion rates with a little reduction of yields for the third run (Figure 6).

FTIR (cm^−1^): 3,359, 3,041 (Ar–H stretching), 2,958, 2,871 (C–H stretching of alkanes), 1,631 (C=N), 1,503 (C=C), 1,154 (C-N).

^1^HNMR (400 MHz, D_2_O) δ (ppm): 0.85 (3H, CH_3_), 1.33 (2H, CH_2_), 1.9 (2H, CH_2_), 2.46 (3H, CH_3_), 4.46 (2H, CH_2_), 7.9 (1H, Ar–H), 8.27 (1H, Ar–H), 8.62 (1H, Ar–H), 8.67 (1H, Ar–H).

## Conclusion

IL-based catalysts containing [BMPy]Cl coupled with nine different metal chlorides have been applied for effective deconstruction of wheat straw biomass. The generation of TRS was 70%, which accounted for 32% conversion of carbohydrate contents of wheat straw when 10 wt% CoCl_2_·6H_2_O was used with PyIL at 100°C for 2 h with 100 μm wheat straw particle size. In addition, 67 wt% lignin was separated from wheat straw by the same catalytic system. In this system, the percentage conversion of wheat straw carbohydrates content is somehow comparable to that of pure cellulose, indicating that other components, especially lignin, have little effect on carbohydrate conversion. Delignification and refinement of cellulose in regenerated material were confirmed by FTIR, SEM, and XRD analyses. The IL–metal catalytic systems showed greater regeneration (86% recovery for [BMPy]^+^${\text{CoCl}}_{3}^{-}$) and recycling abilities. Thus, these IL-based catalytic systems were found to be sustainable for conversion of wheat straw to valuable products.

## Data Availability Statement

The raw data supporting the conclusions are provided in this article and its supplementary information.

## Author Contributions

SN performed the experimental work and wrote the paper. MU supervised the work and corrected the manuscript. AA, NM, and FS have partially written the paper.

### Conflict of Interest

The authors declare that the research was conducted in the absence of any commercial or financial relationships that could be construed as a potential conflict of interest.

## References

[B1] Abou-Yousef H. Steele P. (2013). Rapid conversion of cellulose to 5-hydroxymethylfurfural using single and combined metal chloride catalysts in ionic liquid. J. Fuel Chem. Technol. 41, 214–222. 10.1016/S1872-5813(13)60013-4

[B2] Alinia R. Zabihi S. Esmaeilzadeh F. Kalajahi J. F. (2010). Pretreatment of wheat straw by supercritical CO_2_ and its enzymatic hydrolysis for sugar production. Biosyst. Eng. 107, 61–66. 10.1016/j.biosystemseng.2010.07.002

[B3] Alvira P. Tomas-Pejo E. Ballesteros M. J. Negro M. J. (2010). Pretreatment technologies for an efficient bioethanol production process based on enzymatic hydrolysis: a review. Bioresour. Technol. 101, 4851–4861. 10.1016/j.biortech.2009.11.093 20042329

[B4] Asim A. M. Uroos M. Naz S. Sultan M. Griffin G. Muhammad N. (2019). Acidic ionic liquids: promising and cost-effective solvents for processing of lignocellulosic biomass. J. Mol. Liq. 287, 110943–111109. 10.1016/j.molliq.2019.110943

[B5] Binder J. B. Raines R. T. (2009). Simple chemical transformation of lignocellulosic biomass into furans for fuels and chemicals. J. Am. Chem. Soc. 131, 1979–1985. 10.1021/ja808537j 19159236

[B6] Bozell J. J. Petersen G. R. (2010). Technology development for the production of biobased products from biorefinery carbohydrates—the US Department of Energy's “Top 10” revisited. Green Chem. 12, 539–554. 10.1039/b922014c

[B7] Brandt A. Grasvik J. Hallett J. P. Welton T. (2013). Deconstruction of lignocellulosic biomass with ionic liquids. Green Chem. 15, 550–583. 10.1039/c2gc36364j

[B8] Chambon C. L. Mkhize T. Y. Reddy P. Brandt-Talbot A. Deenadayalu N. Fennell P. S.. (2018). Pretreatment of South African sugarcane bagasse using a low-cost protic ionic liquid: a comparison of whole, depithed, fibrous and pith bagasse fractions. Biotechnol. Biofuels 11:247. 10.1186/s13068-018-1247-0 30214485 PMC6131805

[B9] Chen T. Xiong C. Tao Y. (2018). Enhanced hydrolysis of cellulose in ionic liquid using mesoporous ZSM-5. Molecules 23, 529–539. 10.3390/molecules23030529 29495459 PMC6017767

[B10] Cheng J. Wang N. Zhao D. Qin D. Si W. Tan Y.. (2016). The enhancement of the hydrolysis of bamboo biomass in ionic liquid with chitosan-based solid acid catalysts immobilized with metal ions. Bioresour. Technol. 220, 457–463. 10.1016/j.biortech.2016.08.064 27611029

[B11] da Costa Lopes A. M. Lins R. M. G. Rebelo R. A. Lukasik R.M. (2018). Biorefinery approach for lignocellulosic biomass valorisation with acidic ionic liquid. Green Chem. 17, 4034–4057. 10.1039/C8GC01763H

[B12] da Silva A. S. Inoue H. Endo T. Yano S. Bon E. P. (2010). Milling pretreatment of sugarcane bagasse and straw for enzymatic hydrolysis and ethanol fermentation. Bioresour. Technol. 101, 7402–7409. 10.1016/j.biortech.2010.05.008 20576565

[B13] Eminov S. Brandt A. Wilton-Ely J. D. E. T. Hallett J. P. (2016). The highly selective and near-quantitative conversion of glucose to 5-hydroxymethylfurfural using ionic liquids. PLoS ONE 11:e0163835. 10.1371/journal.pone.0163835 27711238 PMC5053443

[B14] Gschwend F. J. V. Chambon C. L. Biedka M. Brandt A. Fennell P. S. Hallett J. P. (2012). Quantitative glucose release from softwood after pretreatment with low-cost ionic liquids. Green Chem. 21, 692–703. 10.1039/C8GC02155D

[B15] Heinze T. Schwikal K. Barthel S. (2005). Ionic liquids as reaction medium in cellulose functionalization. Macromol. Biosci. 5, 520–525. 10.1002/mabi.200500039 15948229

[B16] Hsu T.-C. Guo G.-L. Chen W.-H. Hwang W.-S. (2010). Effect of dilute acid pretreatment of rice straw on structural properties and enzymatic hydrolysis. Bioresour. Technol. 101, 4907–4913. 10.1016/j.biortech.2009.10.009 19926476

[B17] Kassaye S. Pant K. K. Jain S. (2017). Hydrolysis of cellulosic bamboo biomass into reducing sugars via a combined alkaline solution and ionic liquid pretreament steps. Renew. Ener. 104, 177–184. 10.1016/j.renene.2016.12.033

[B18] Ma F. Yang N. Xu C. Yu H. Wu J. Zhang X. (2010). Combination of biological pretreatment with mild acid pretreatment for enzymatic hydrolysis and ethanol production from water hyacinth. Bioresour. Technol. 101, 9600–9604. 10.1016/j.biortech.2010.07.084 20705458

[B19] Mosier N. Wyman C. Dale B. Elander R. Lee Y. Y. Holtzapple M.. (2005). Features of promising technologies for pretreatment of lignocellulosic biomass. Bioresour. Technol. 96, 673–686. 10.1016/j.biortech.2004.06.025 15588770

[B20] Moyer P. Smith M. D. Abdoulmoumine N. Chmely S. C. Smith J. C. Petridis L.. (2018). Relationship between lignocellulosic biomass dissolution and physicochemical properties of ionic liquids composed of 3-methylimidazolium cations and carboxylate anions. Phys. Chem. Chem. Phys. 20, 2508–2516. 10.1039/C7CP07195G 29313537

[B21] Ogura K. Ninomiya K. Takahashi K. Ogino C. Kondo A. (2014). Pretreatment of Japanese cedar by ionic liquid solutions in combination with acid and metal ion and its application to high solid loading. Biotechnol. Biofuels 7:120. 10.1186/s13068-014-0120-z 25426161 PMC4243821

[B22] Pang Z. Dong C. Pan X. (2016). Enhanced deconstruction and dissolution of lignocellulosic biomass in ionic liquid at high water content by lithium chloride. Cellulose 23, 323–338. 10.1007/s10570-015-0832-7

[B23] Plazonic I. Barbaric-Mikocevic Z. Antonovic A. (2016). Chemical composition of straw as an alternative material to wood raw material in fibre isolation. Drv. Ind. 67, 119–125. 10.5552/drind.2016.1446

[B24] Qu Y. Li L. Wei Q. Huang C. Oleskowicz-Popiel P. Xu J. (2016). One-pot conversion of disaccharide into 5-hydroxymethylfurfural catalyzed by imidazole ionic liquid. Sci. Rep. 6:26067. 10.1038/srep26067 27181523 PMC4867639

[B25] Ramli N. A. S. Amin N. A. S. (2014). Catalytic hydrolysis of cellulose and oil palm biomass in ionic liquid to reducing sugar for levulinic acid production. Fuel Process. Technol. 128, 490–498. 10.1016/j.fuproc.2014.08.011

[B26] Rocha E. G. A. Pin T. C. Rabelo S. C. Costa A. C. (2017). Evaluation of the use of protic ionic liquids on biomass fractionation. Fuel 206, 145–154. 10.1016/j.fuel.2017.06.014

[B27] Saher S. Saleem H. Asim A. M. Uroos M. Muhammad N. (2018). Pyridinium-based ionic liquid: a pretreatment solvent and reaction medium for catalytic conversion of cellulose to total reducing sugars (TRS). J. Mol. Liq. 272, 330–336. 10.1016/j.molliq.2018.09.099

[B28] Sashina E. S. Kashirskii D. A. Busygin K. N. (2016). Dissolution of cellulose with pyridinium-based ionic liquids: effect of chemical structure and interaction mechanism. *Cellulose Chem*. Technol. 50, 199–211.

[B29] Semerci I. Guler F. (2018). Protic ionic liquids as effective agents for pretreatment of cotton stalks at high biomass loading. Ind. Crops Prod. 125, 588–595. 10.1016/j.indcrop.2018.09.046

[B30] Sievers C. Valenzuela-Olarte M. B. Marzialetti T. Musin I. Agrawal P. K. Jones C. W. (2009). Ionic-liquid-phase hydrolysis of pine wood. Ind. Eng. Chem. Res. 48, 1277–1286. 10.1021/ie801174x

[B31] Song J. Zhang B. Shi J. Fan H. Ma J. Yang Y. (2013). Efficient conversion of glucose and cellulose to 5-hydroxymethylfurfural in DBU-based ionic liquids. RSC Adv. 3, 20085–20090. 10.1039/c3ra43934h

[B32] Sun N. Rahman M. Qin Y. Maxim M. L. Rodríguez H. Rogers R. D. (2009). Complete dissolution and partial delignification of wood in the ionic liquid 1-ethyl-3-methylimidazolium acetate. Green Chem. 11, 646–655. 10.1039/b822702k

[B33] Suzuki S. Takeoka Y. Rikukawa M. Fujita M. Y. (2018). Bronsted acidic ionic liquids for cellulose hydrolysis in an aqueous medium: structural effects on acidity and glucose yield. RSC Adv. 8, 14623–14632. 10.1039/C8RA01950A PMC9079954 35540788

[B34] Tao F. Song H. Chou L. (2010). Hydrolysis of cellulose by using catalytic amounts of FeCl_2_ in ionic liquids. ChemSusChem 3, 1298–1303. 10.1002/cssc.201000184 20936646

[B35] Tao F. Song H. Chou L. (2011). Catalytic conversion of cellulose to chemicals in ionic liquid. Carbohydr. Res. 346, 58–63. 10.1016/j.carres.2010.10.022 21092940

[B36] Varga E. Reczey K. Zacchi G. (2004). “Optimization of steam pretreatment of corn stover to enhance enzymatic digestibility,” in Proceedings of the Twenty-Fifth Symposium on Biotechnology for Fuels and Chemicals Held May 4–7 2003, (Breckenridge, CO:Springer), 509–523. 10.1007/978-1-59259-837-3_43 15054274

[B37] Wan C. Li Y. (2010). Microbial pretreatment of corn stover with Ceriporiopsis subvermispora for enzymatic hydrolysis and ethanol production. Bioresour. Technol. 101, 6398–6403. 10.1016/j.biortech.2010.03.070 20381341

[B38] Wang N. Zhang J. Wang H. Li Q. Wang D. (2014). Effects of metal ions on the hydrolysis of bamboo biomass in 1-butyl-3-methylimidazolium chloride with dilute acid as catalyst. Bioresour. Technol. 173, 399–405. 10.1016/j.biortech.2014.09.125 25444883

[B39] Wang P. Yu H. Zhan S. Wang S. (2011). Catalytic hydrolysis of lignocellulosic biomass into 5-hydroxymethylfurfural in ionic liquid. Bioresour. Technol. 102, 4179–4183. 10.1016/j.biortech.2010.12.073 21232942

[B40] Weerachanchai P. Lee J.-M. (2013). Effect of organic solvent in ionic liquid on biomass pretreatment. ACS Sustain. Chem. Eng. 1, 894–902. 10.1021/sc300147f

[B41] Xiao S. Liu B. Wang Y. Fang Z. Zhang Z. (2014). Efficient conversion of cellulose into biofuel precursor 5-hydroxymethylfurfural in dimethyl sulfoxide–ionic liquid mixtures. Bioresour. Technol. 151, 361–366. 10.1016/j.biortech.2013.10.095 24269827

[B42] Xu F. Sun J. Konda N. M. Shi J. Dutta T. Scown C. D. (2016). Transforming biomass conversion with ionic liquids: process intensification and the development of a high-gravity, one-pot process for the production of cellulosic ethanol. Ener. Environ. Sci. 3, 1042–1049. 10.1039/C5EE02940F

[B43] Yoo C. G. Pu Y. Ragauskas A. J. (2017). Ionic liquids: promising green solvents for lignocellulosic biomass utilization. Curr. Opin. Green Sustain. Chem. 5, 5–11. 10.1016/j.cogsc.2017.03.003

[B44] Yu X. Baoa X. Zhoua C. Zhanga L. Yagoubb A. A. Yanga H.. (2018). Ultrasound-ionic liquid enhanced enzymatic and acid hydrolysis of biomass cellulose. Ultrason. Sonochem. 41, 410–418. 10.1016/j.ultsonch.2017.09.003 29137769

[B45] Yuan Y. Yao S. Nie S. Wang S. (2016). Conversion of glucose into HMF catalyzed by CPL-LiCl investigated using dual-wavelength UV spectrophotometry. BioResources 11, 2381–2392. 10.15376/biores.11.1.2381-2392

[B46] Zakzeski J. Bruijnincx P. C. Jongerius A. L. Weckhuysen B. M. (2010). The catalytic valorization of lignin for the production of renewable chemicals. Chem. Rev. 110, 3552–3599. 10.1021/cr900354u 20218547

[B47] Zhang Z. Song J. Han B. (2016). Catalytic transformation of lignocellulose into chemicals and fuel products in ionic liquids. Chem. Rev. 117, 6834–6880. 10.1021/acs.chemrev.6b00457 28535680

[B48] Zhang Z. Zhao Z. K. (2010). Microwave-assisted conversion of lignocellulosic biomass into furans in ionic liquid. Bioresour. Technol. 101, 1111–1114. 10.1016/j.biortech.2009.09.010 19800219

[B49] Zhao H. Holladay J. E. Brown H. Zhang Z. C. (2007). Metal chlorides in ionic liquid solvents convert sugars to 5-hydroxymethylfurfural. Science 316, 1597–1600. 10.1126/science.1141199 17569858

[B50] Zhou L. He Y. Ma Z. Liang R. Wu T. Wu Y. (2015). One-step degradation of cellulose to 5-hydroxymethylfurfural in ionic liquid under mild conditions. Carbohydr. Polym. 117, 694–700. 10.1016/j.carbpol.2014.10.062 25498690

[B51] Zhou L. Liang R. Ma Z. Wu T. Wu Y. (2013). Conversion of cellulose to HMF in ionic liquid catalyzed by bifunctional ionic liquids. Bioresour. Technol. 129, 450–455. 10.1016/j.biortech.2012.11.015 23266845

[B52] Zhu S. Wu Y. Yu Z. Chen Q. Wu G. Yu F. (2006). Microwave-assisted alkali pre-treatment of wheat straw and its enzymatic hydrolysis. Biosyst. Eng. 94, 437–442. 10.1016/j.biosystemseng.2006.04.002

