# Supplementary material for: One-Pot Deconstruction and Conversion of Lignocellulose Into Reducing Sugars by Pyridinium-Based Ionic Liquid–Metal Salt System
Source: Front Chem. 2020 Apr 15;8:236. doi: 10.3389/fchem.2020.00236 (PMC7174784; doi:10.3389/fchem.2020.00236)
Supplement: Supplementary file 1 [file Data_Sheet_1.docx]

**Supporting information**

**One-Pot Deconstruction and Conversion of Lignocellulose into Reducing Sugars by Pyridinium Based Ionic Liquid‒Metal Salt System**

**Sadia Naz ^a^, Maliha Uroos ^a, *^, Azmat Mehmood Asim ^a^, Nawshad Muhammad ^b,^ and Faiz Ullah Shah ^c^,^*^**

^a^ Institute of Chemistry, University of the Punjab, 54000, Lahore, Pakistan

^b^ Interdisciplinary Research Centre in Biomedical Materials (IRCBM), COMSATS University Islamabad, Lahore Campus, 54600, Lahore, Pakistan

^c^ Chemistry of Interfaces, Luleå University of Technology, 971 87 Luleå, Sweden

^*^Corresponding authors: [malihauroos.chem@pu.edu.pk](mailto:malihauroos.chem@pu.edu.pk); and [faiz.ullah@ltu.se](mailto:faiz.ullah@ltu.se)

**Fig. S1.** Standard DNS curve for glucose concentration.


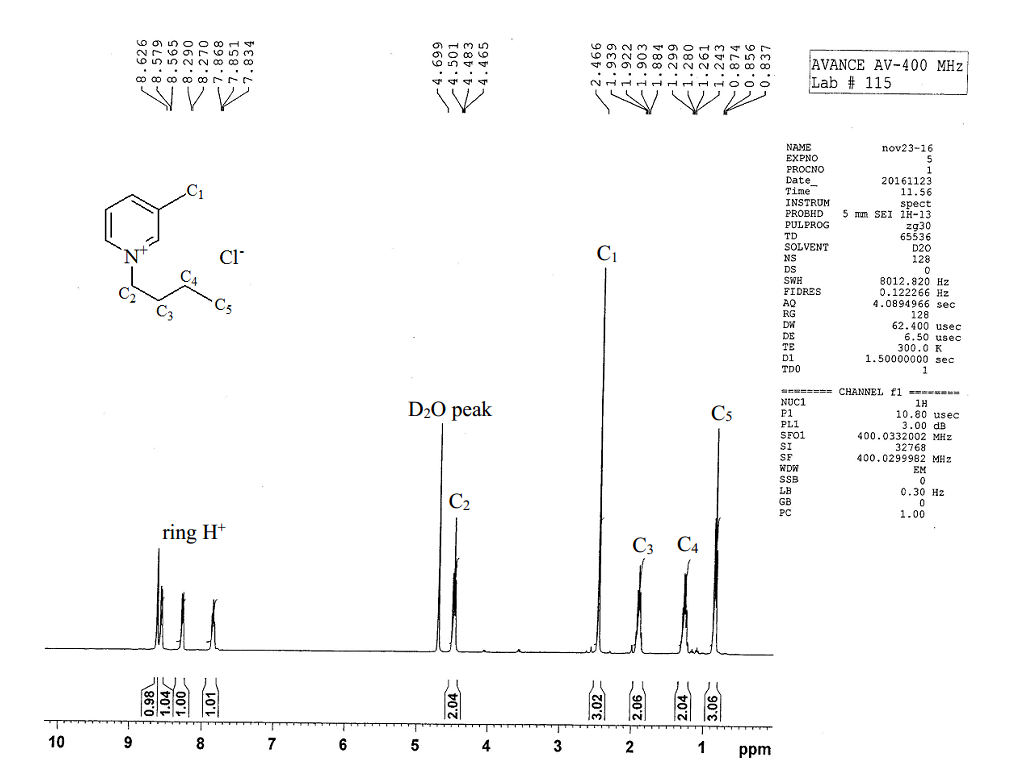


**Fig. S2.** ^1^HNMR of [BMPy]Cl.


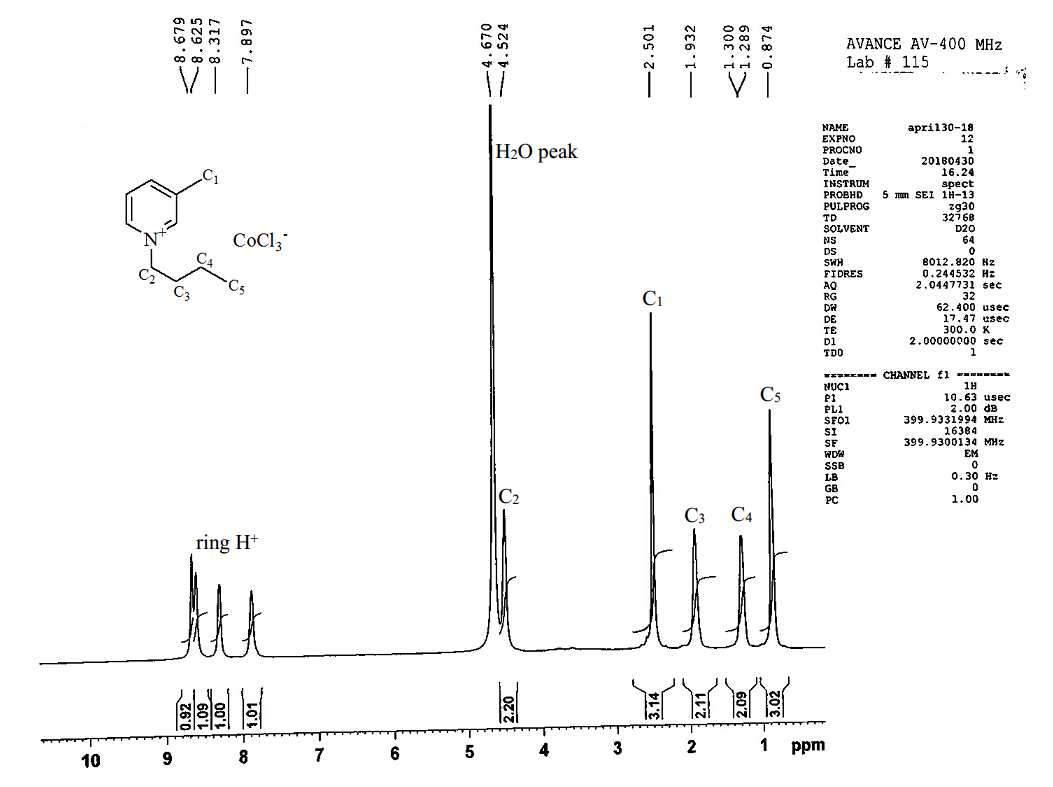


**Fig. S3.** ^1^HNMR of regenerated [BMPy]Cl.

**Fig. S4.** Effect of different metal chlorides on delignified wheat straw/cellulose rich material (CRM).

**Fig. S5.** Effect of different metal chlorides added in IL for lignin extraction.

**Fig. S6.** FTIR analysis of originally synthesized and regenerated [BMPy]Cl.

**Table S1.** Effect of different process variables on one-pot conversion and deconstruction of wheat straw and their analysis of variance (ANOVA) at 0.05 level of confidence showing that X_1_, X_2_, X_3_ and X_4_ are not significantly different.

| **Sr. No.** | **Catalyst loading (%)** | **Time**  **(h)** | **Temp.**  **(°C)** | **Biomass size (μm)** | **TRS**  **(%)** | **% Conversion** | **% Delignification** |
| --- | --- | --- | --- | --- | --- | --- | --- |
|  | **X_1_** | **X_2_** | **X_3_** | **X_4_** |  |  |  |
| 1 | 1 | 2.0 | 100 | 100 | 37 | 12 | 8 |
| 2 | 2 | 2.0 | 100 | 100 | 43 | 12 | 8 |
| 3 | 3 | 2.0 | 100 | 100 | 44 | 12 | 15 |
| 4 | 5 | 2.0 | 100 | 100 | 45 | 15 | 39 |
| 5 | 7 | 2.0 | 100 | 100 | 47 | 20 | 57 |
| 6 | 10 | 2.0 | 100 | 100 | 70 | 32 | 67 |
| 7 | 13 | 2.0 | 100 | 100 | 16 | 14 | 65 |
| 8 | 16 | 2.0 | 100 | 100 | 55 | 15 | 54 |
| 9 | 10 | 0.5 | 100 | 100 | 41 | 17 | 20 |
| 10 | 10 | 1.0 | 100 | 100 | 65 | 25 | 45 |
| 11 | 10 | 1.5 | 100 | 100 | 66 | 31 | 64 |
| 12 | 10 | 2.5 | 100 | 100 | 63 | 30 | 65 |
| 13 | 10 | 3.0 | 100 | 100 | 62 | 20 | 59 |
| 14 | 10 | 2.0 | 80 | 100 | 29 | 12 | 62 |
| 15 | 10 | 2.0 | 120 | 100 | 71 | 15 | 40 |
| 16 | 10 | 2.0 | 100 | 250 | 60 | 27 | 65 |
| 17 | 10 | 2.0 | 100 | 500 | 39 | 15 | 60 |
| 18 | 10 | 2.0 | 100 | 100 | 62 | 19 | 54 |
| 19 | 10 | 2.0 | 100 | 100 | 40 | 5 | 47 |

| **ANOVA** | | | | | | | | | | | | | | | | | | | | | | |
| --- | --- | --- | --- | --- | --- | --- | --- | --- | --- | --- | --- | --- | --- | --- | --- | --- | --- | --- | --- | --- | --- | --- |
| **Variable X_1_** | | | | | | | | Sum of Squares | | | df | | | Mean Square | | | F | | | Sig. value | | |
| TRS  R^2^ = 0.4531 | Between Groups | | | (Combined) | | | | 1853.018 | | | 7 | | | 264.717 | | | 1.302 | | | .334 | | |
|  |  |  |  | Linear Term | | Weighted | | 136.680 | | | 1 | | | 136.680 | | | .672 | | | .430 | | |
|  |  |  |  |  |  | Deviation | | 1716.337 | | | 6 | | | 286.056 | | | 1.407 | | | .295 | | |
|  |  |  |  | Quadratic Term | | Weighted | | 285.721 | | | 1 | | | 285.721 | | | 1.405 | | | .261 | | |
|  |  |  |  |  |  | Deviation | | 1430.617 | | | 5 | | | 286.123 | | | 1.407 | | | .295 | | |
|  | Within Groups | | | | | | | 2236.667 | | | 11 | | | 203.333 | | |  | | |  | | |
|  | Total | | | | | | | 4089.684 | | | 18 | | |  | | |  | | |  | | |
| Conversion  R^2^ = 0.22669 | Between Groups | | | (Combined) | | | | 229.439 | | | 7 | | | 32.777 | | | .461 | | | .844 | | |
|  |  |  |  | Linear Term | | Weighted | | 69.215 | | | 1 | | | 69.215 | | | .973 | | | .345 | | |
|  |  |  |  |  |  | Deviation | | 160.224 | | | 6 | | | 26.704 | | | .375 | | | .880 | | |
|  |  |  |  | Quadratic Term | | Weighted | | 121.760 | | | 1 | | | 121.760 | | | 1.711 | | | .217 | | |
|  |  |  |  |  |  | Deviation | | 38.464 | | | 5 | | | 7.693 | | | .108 | | | .988 | | |
|  | Within Groups | | | | | | | 782.667 | | | 11 | | | 71.152 | | |  | | |  | | |
|  | Total | | | | | | | 1012.105 | | | 18 | | |  | | |  | | |  | | |
| Delignification  R^2^ = 0.71022 | Between Groups | | | (Combined) | | | | 5190.947 | | | 7 | | | 741.564 | | | 3.851 | | | .023 | | |
|  |  |  |  | Linear Term | | Weighted | | 4031.910 | | | 1 | | | 4031.910 | | | 20.940 | | | .001 | | |
|  |  |  |  |  |  | Deviation | | 1159.037 | | | 6 | | | 193.173 | | | 1.003 | | | .470 | | |
|  |  |  |  | Quadratic Term | | Weighted | | 862.879 | | | 1 | | | 862.879 | | | 4.481 | | | .058 | | |
|  |  |  |  |  |  | Deviation | | 296.159 | | | 5 | | | 59.232 | | | .308 | | | .898 | | |
|  | Within Groups | | | | | | | 2118.000 | | | 11 | | | 192.545 | | |  | | |  | | |
|  | **Total** | | | | | | | 7308.947 | | | 18 | | |  | | |  | | |  | | |
| **Variable X_2_** | | | | | | | | | | | | | | | | | | | | | | |
| TRS  R^2^ = 0.24444 | | | Between Groups | | (Combined) | | | | | 999.684 | | | 5 | | | 199.937 | | .841 | | | .544 | |
|  |  |  |  |  | Linear Term | | Unweighted | | | 92.658 | | | 1 | | | 92.658 | | .390 | | | .543 | |
|  |  |  |  |  |  |  | Weighted | | | 19.056 | | | 1 | | | 19.056 | | .080 | | | .782 | |
|  |  |  |  |  |  |  | Deviation | | | 980.628 | | | 4 | | | 245.157 | | 1.031 | | | .428 | |
|  |  |  |  |  | Quadratic Term | | Unweighted | | | 61.105 | | | 1 | | | 61.105 | | .257 | | | .621 | |
|  |  |  |  |  |  |  | Weighted | | | 87.449 | | | 1 | | | 87.449 | | .368 | | | .555 | |
|  |  |  |  |  |  |  | Deviation | | | 893.179 | | | 3 | | | 297.726 | | 1.253 | | | .331 | |
|  |  |  | Within Groups | | | | | | | 3090.000 | | | 13 | | | 237.692 | |  | | |  | |
|  |  |  | Total | | | | | | | 4089.684 | | | 18 | | |  | |  | | |  | |
| Conversion  R^2^ = 0.41219 | | | Between Groups | | (Combined) | | | | | 417.177 | | | 5 | | | 83.435 | | 1.823 | | | .177 | |
|  |  |  |  |  | Linear Term | | Unweighted | | | 3.289 | | | 1 | | | 3.289 | | .072 | | | .793 | |
|  |  |  |  |  |  |  | Weighted | | | 2.685 | | | 1 | | | 2.685 | | .059 | | | .812 | |
|  |  |  |  |  |  |  | Deviation | | | 414.492 | | | 4 | | | 103.623 | | 2.264 | | | .118 | |
|  |  |  |  |  | Quadratic Term | | Unweighted | | | 49.133 | | | 1 | | | 49.133 | | 1.074 | | | .319 | |
|  |  |  |  |  |  |  | Weighted | | | 20.936 | | | 1 | | | 20.936 | | .457 | | | .511 | |
|  |  |  |  |  |  |  | Deviation | | | 393.556 | | | 3 | | | 131.185 | | 2.867 | | | .077 | |
|  |  |  | Within Groups | | | | | | | 594.929 | | | 13 | | | 45.764 | |  | | |  | |
|  |  |  | Total | | | | | | | 1012.105 | | | 18 | | |  | |  | | |  | |
| Delignification  R^2^ = 0.20668 | | | Between Groups | | (Combined) | | | | | 1510.590 | | | 5 | | | 302.118 | | .677 | | | .648 | |
|  |  |  |  |  | Linear Term | | Unweighted | | | 811.732 | | | 1 | | | 811.732 | | 1.820 | | | .200 | |
|  |  |  |  |  |  |  | Weighted | | | 655.001 | | | 1 | | | 655.001 | | 1.469 | | | .247 | |
|  |  |  |  |  |  |  | Deviation | | | 855.589 | | | 4 | | | 213.897 | | .480 | | | .750 | |
|  |  |  |  |  | Quadratic Term | | Unweighted | | | 343.637 | | | 1 | | | 343.637 | | .770 | | | .396 | |
|  |  |  |  |  |  |  | Weighted | | | 10.653 | | | 1 | | | 10.653 | | .024 | | | .880 | |
|  |  |  |  |  |  |  | Deviation | | | 844.936 | | | 3 | | | 281.645 | | .631 | | | .608 | |
|  |  |  | Within Groups | | | | | | | 5798.357 | | | 13 | | | 446.027 | |  | | |  | |
|  |  |  | **Total** | | | | | | | 7308.947 | | | 18 | | |  | |  | | |  | |
| **Variable X3** | | | | | | | | | | | | | | | | | | | | | | |
| TRS  R^2^ = 1 | | Between Groups | | | (Combined) | | | | | 882.155 | | | 2 | | | 441.077 | | 2.200 | | | .143 | |
|  |  |  |  |  | Linear Term | | Unweighted | | | 882.000 | | | 1 | | | 882.000 | | 4.400 | | | .052 | |
|  |  |  |  |  |  |  | Weighted | | | 882.000 | | | 1 | | | 882.000 | | 4.400 | | | .052 | |
|  |  |  |  |  |  |  | Deviation | | | .155 | | | 1 | | | .155 | | .001 | | | .978 | |
|  |  |  |  |  | Quadratic Term | | Unweighted | | | .155 | | | 1 | | | .155 | | .001 | | | .978 | |
|  |  |  |  |  |  |  | Weighted | | | .155 | | | 1 | | | .155 | | .001 | | | .978 | |
|  |  | Within Groups | | | | | | | | 3207.529 | | | 16 | | | 200.471 | |  | | |  | |
|  |  | Total | | | | | | | | 4089.684 | | | 18 | | |  | |  | | |  | |
| Conversion  R^2^ = 0.05567 | | Between Groups | | | (Combined) | | | | | 56.341 | | | 2 | | | 28.170 | | .472 | | | .632 | |
|  |  |  |  |  | Linear Term | | Unweighted | | | 4.500 | | | 1 | | | 4.500 | | .075 | | | .787 | |
|  |  |  |  |  |  |  | Weighted | | | 4.500 | | | 1 | | | 4.500 | | .075 | | | .787 | |
|  |  |  |  |  |  |  | Deviation | | | 51.841 | | | 1 | | | 51.841 | | .868 | | | .365 | |
|  |  |  |  |  | Quadratic Term | | Unweighted | | | 51.841 | | | 1 | | | 51.841 | | .868 | | | .365 | |
|  |  |  |  |  |  |  | Weighted | | | 51.841 | | | 1 | | | 51.841 | | .868 | | | .365 | |
|  |  | Within Groups | | | | | | | | 955.765 | | | 16 | | | 59.735 | |  | | |  | |
|  |  | Total | | | | | | | | 1012.105 | | | 18 | | |  | |  | | |  | |
| Delignification  R^2^ = 0.03788 | | Between Groups | | | (Combined) | | | | | 276.830 | | | 2 | | | 138.415 | | .315 | | | .734 | |
|  |  |  |  |  | Linear Term | | Unweighted | | | 242.000 | | | 1 | | | 242.000 | | .551 | | | .469 | |
|  |  |  |  |  |  |  | Weighted | | | 242.000 | | | 1 | | | 242.000 | | .551 | | | .469 | |
|  |  |  |  |  |  |  | Deviation | | | 34.830 | | | 1 | | | 34.830 | | .079 | | | .782 | |
|  |  |  |  |  | Quadratic Term | | Unweighted | | | 34.830 | | | 1 | | | 34.830 | | .079 | | | .782 | |
|  |  |  |  |  |  |  | Weighted | | | 34.830 | | | 1 | | | 34.830 | | .079 | | | .782 | |
|  |  | Within Groups | | | | | | | | 7032.118 | | | 16 | | | 439.507 | |  | | |  | |
|  |  | **Total** | | | | | | | | 7308.947 | | | 18 | | |  | |  | | |  | |
| **Variable X_4_** | | | | | | | | | | | | | | | | | | | | | | |
| TRS  R^2^ = 0.05423 | | Between Groups | | (Combined) | | | | | 221.802 | | | 2 | | | 110.901 | | | | .459 | | | .640 |
|  |  |  |  | Linear Term | | Weighted | | | 55.652 | | | 1 | | | 55.652 | | | | .230 | | | .638 |
|  |  |  |  |  |  | Deviation | | | 166.150 | | | 1 | | | 166.150 | | | | .687 | | | .419 |
|  |  |  |  | Quadratic Term | | Weighted | | | 166.150 | | | 1 | | | 166.150 | | | | .687 | | | .419 |
|  |  | Within Groups | | | | | | | 3867.882 | | | 16 | | | 241.743 | | | |  | | |  |
|  |  | Total | | | | | | | 4089.684 | | | 18 | | |  | | | |  | | |  |
| Conversion  R^2^ = 0.08705 | | Between Groups | | (Combined) | | | | | 88.105 | | | 2 | | | 44.053 | | | | .763 | | | .483 |
|  |  |  |  | Linear Term | | Weighted | | | .003 | | | 1 | | | .003 | | | | .000 | | | .994 |
|  |  |  |  |  |  | Deviation | | | 88.102 | | | 1 | | | 88.102 | | | | 1.526 | | | .235 |
|  |  |  |  | Quadratic Term | | Weighted | | | 88.102 | | | 1 | | | 88.102 | | | | 1.526 | | | .235 |
|  |  | Within Groups | | | | | | | 924.000 | | | 16 | | | 57.750 | | | |  | | |  |
|  |  | Total | | | | | | | 1012.105 | | | 18 | | |  | | | |  | | |  |
| Delignification  R^2^ = 0.07469 | | Between Groups | | (Combined) | | | | | 545.889 | | | 2 | | | 272.944 | | | | .646 | | | .537 |
|  |  |  |  | Linear Term | | Weighted | | | 371.917 | | | 1 | | | 371.917 | | | | .880 | | | .362 |
|  |  |  |  |  |  | Deviation | | | 173.972 | | | 1 | | | 173.972 | | | | .412 | | | .530 |
|  |  |  |  | Quadratic Term | | Weighted | | | 173.972 | | | 1 | | | 173.972 | | | | .412 | | | .530 |
|  |  | Within Groups | | | | | | | 6763.059 | | | 16 | | | 422.691 | | | |  | | |  |
|  |  | **Total** | | | | | | | 7308.947 | | | 18 | | |  | | | |  | | |  |
